# Intimate partner violence reported by female and male users of healthcare units

**DOI:** 10.1590/S1518-8787.2017051006385

**Published:** 2017-02-08

**Authors:** Claudia Renata dos Santos Barros, Lilia Blima Schraiber

**Affiliations:** I Universidade Católica de Santos. Santos, SP, Brasil; II Departamento de Medicina Preventiva. Faculdade de Medicina. Universidade de São Paulo. São Paulo, SP, Brasil

**Keywords:** Intimate Partner Violence, Domestic Violence, Spouse Abuse, Gender and Health

## Abstract

**OBJECTIVE:**

To analyze nonfatal violence suffered and committed by adult men and women, in an intimate relationship.

**METHODS:**

The participants in the research were women aged between 15 and 49 years and men between 18 and 60 years, interviewed by face-to-face questionnaire application. The sample selection was of consecutive type, according to the order of arrival of the users. We conducted temporarily independent investigations and covered different health services to avoid couples and relationships in which the retaliation could be overvalued. To improve the comparison, we also examined reports of men and women from the same service, i.e., a service that was common to both investigations. We compared the situations suffered by women according to their reports and cross-linked the information to what men, according to their own reports, do against intimate partners or ex-partners. We also examined the cross-linked situation in reverse: the violence committed by women against their partners, according to their reports, in comparison with the violence suffered by men, also according to their reports, even if, in this case, the exam refers only to physical violence. The variables were described using mean, standard deviation, frequencies and proportions, and the hypothesis testing used was: Fisher’s exact and Pearson’s Chi-square tests, adopting a significance level of 5%.

**RESULTS:**

Victimization was greater among women, regardless of the type of violence, when perpetrated by intimate partner. The perception of violence was low in both genders; however, women reported more episodes of multiple recurrences of any violence and sexual abuse suffered than men acknowledged to have perpetrated.

**CONCLUSIONS:**

The study in its entirety shows significant gender differences, whether about the prevalence of violence, whether about the perception of these situations.

## RESUMO

**OBJETIVO:**

Analisar as violências não fatais sofridas e praticadas por homens e mulheres adultos, em situação de parceria íntima.

**MÉTODOS:**

Os sujeitos da pesquisa foram mulheres entre 15 a 49 anos e homens entre 18 a 60 anos, entrevistados por aplicação de questionário face a face. A seleção amostral foi do tipo consecutivo, captando-se os participantes por ordem de chegada ao serviço. Foram conduzidos como investigações temporalmente independentes e abrangendo diferentes serviços, para evitar o estudo em casais, nos quais o revide poderia estar supervalorizado. Para adensar um pouco mais a comparação, também examinamos relatos de homens e mulheres pertencentes a um mesmo serviço, ou seja, um serviço que foi participante comum às duas investigações. Comparamos as situações sofridas pelas mulheres segundo seus relatos e, de modo entrecruzado, as situações que os homens, segundo seus relatos, praticam contra as mulheres, suas parceiras íntimas ou ex-parceiras. Também examinamos a situação entrecruzada reversa: a violência praticada pelas mulheres contra seus parceiros, segundo seus próprios relatos, comparativamente à violência sofrida pelos homens, segundo seus relatos, ainda que, neste caso, o exame se refere apenas à violência física. As variáveis foram descritas por meio de média, desvio padrão, frequências e proporções e os testes de hipóteses utilizados foram: Exato de Fisher e Qui-quadrado de Pearson, adotando-se nível de significância de 5%.

**RESULTADOS:**

A vitimização foi maior entre as mulheres, independentemente do tipo de violência, quando perpetrada por parceiro íntimo. A percepção da violência foi baixa em ambos os sexos; entretanto, mulheres relataram mais episódios de múltiplas recorrências de quaisquer violências e de violência sexual sofrida do que os homens reconheceram que perpetraram.

**CONCLUSÕES:**

O estudo em seu todo mostra importantes diferenças de gênero, quer quanto às prevalências das violências, quer quanto à percepção dessas situações.

## INTRODUCTION

Using the typology of patterns of violence presented by the World Health Organization in the World Report on Violence and Health^1^, we can affirm that, currently, important studies have been produced on nonfatal interpersonal violence for the adult population (18 years or more), represented centrally by research on the violence suffered by women, especially in the reproductive range (15 to 49 years). The studies emphasize the domestic character of this type of violence, as the main aggressor was always the intimate partner^2,3^.

Surveys that deal with nonfatal interpersonal violence and of domestic origin suffered by men are scarce, being more studied the male condition as an aggressor in domestic violence^4^.

Review study conducted with men and women aged 18 years or more^5^ showed that there are differences in violent situations according to gender – women suffer more physical and sexual violence by their partners. Longitudinal study^6^, investigating the physical violence suffered and perpetrated by men and women over the age of 18 years, found higher prevalence of intimate violence suffered and perpetrated among women, except the perpetration of sexual violence against the partner, which was higher by men. Similar patterns of the occurrences have been shown by Brazilian studies^4,7-9^.

Regarding the distinctions between the violence experienced by men and women, as to who is the main aggressor, what is the type of interpersonal relationship between aggressor and victim, and if the episodes occur more in public or private places, in many countries, the rates of physical violence are more frequent for men than for women and the reverse occurs in the case of sexual violence, whether it is of domestic nature or not^9,10^.

On the other hand, some studies point out a mutuality of aggressions, also analyzed as gender symmetry in intimate relationship violence. These studies had not observed differences between the violence suffered by adult women and men, but equivalent rates^11,12^. However, other studies^5,13^ point out that the difference often lies in the severity of the acts and in the type of violence suffered, thus the symmetry on sexual violence does not exist in the most serious cases of physical violence, being men, in these cases, the main aggressors.

These authors^5,13^ also argue the possibility of comparing studies so diverse methodologically. In this sense, another aspect that we emphasize is the overlap of the types of violence and the condition of aggressor and victim at the same time, whether they are men or women. Such overlap is not always controlled in the studies.

This study aimed to analyze the nonfatal violence suffered and perpetrated by adult men and women, involved in an intimate relationship.

## METHODS

This is a cross-sectional study based on the database produced in two independent investigations with male and female users of public health services in São Paulo, Brazil. The initial goal in both investigations was to estimate the prevalence of intimate partner psychological, physical, and sexual violence against women, according to what women and men reported, with women as victims and men as the perpetrators of violence. Men were also asked about situations they suffered, as for intimate partner violence and for the three types of violence. However, in the approach of women as perpetrators, according to their own reports, given the secondary character of this goal, the question was restricted to the perpetration of physical violence against their partners.

The episodes of violence were reported as occurrence in life and in the last year before the investigation, in terms of unique occurrences and also of overlapping of the three types of violence, the frequency of episodes, and their severity.

Thus, in this study, we compared: the violence suffered by women and men, according to their own reports; the violence perpetrated by women and men, also according to their own reports; and the situations suffered by women according to their reports, cross-linking the data to the reports of men regarding violence committed against their intimate partners or ex-partners.

In addition, we examined the cross-linked situation in reverse: the violence committed by women against their partners, according to their own reports, compared with the violence suffered by men, according to their reports, even if, in this case, the exam refers only to physical violence. The research conducted with women was held between 2001-2002^14^ and with men, between 2002-2003^4^.

Both aforementioned studies were conducted with users of healthcare units of public health services located in the Midwest region of São Paulo city. These two studies were independent and sequential researches, and the one conducted with men had the same design method to be compared with the study conducted with women. The aim was to facilitate the comparison between these two sex subgroups of the same population of health service users, identified in socioeconomic and geopolitical terms of the city. Thus, the comparative exam concerns users of health services of the same part of the city of São Paulo, the Midwest region.

They were conducted as investigations temporarily independent and covered different services to avoid the study with couples, in which the retaliation could be overvalued. To deepen the comparison, we also examined reports of men and women belonging to the same service, i.e., a service that was common to both investigations. This comparison aimed to capture socioeconomic similarities or possible couples among regulars of the same health service.

For both investigations, health care services were selected by convenience sampling, whose criteria were: significant population demand; existence of multidisciplinary staff with ability to host possible cases enabled by research; quality of medical records in handbooks; suitable conditions for the ethical development of research activities; perception of violence as a medical necessity for leadership and staffs; and, in the case of the research with men, to have been elected service for research previously conducted with female users.

The subjects of the research were women aged between 15 and 49 years and men between 18 and 60 years, interviewed by face-to-face questionnaire application. The sample selection was of consecutive type, capturing the participants by order of arrival. We ensured that research would encompass at least a full week of regular functioning of health services, with proportional allocation of sample to the volumes of appointment by day of the week and time period (morning or afternoon).

The sample of women was calculated based on the expected prevalence of domestic violence at least once in a lifetime of 32.0% to detect a variation of 5.0%, 80.0% test power, and confidence level of 95%, reaching a total of 661 users with intimate relationships once in their lifetime^15^. The sample of men is based on the results found in this investigation conducted with women and on population survey data with women in the city of São Paulo, who used the same questionnaire^10^. We obtained a total of 775 men who had an intimate partner once in their lifetime^4^ to achieve: (1) estimates of the prevalence of different forms of current conjugal violence and, consequently, the identification of the percentage of potential aggressors and their sociodemographic characteristics, under a precision of 5.0% between the estimated prevalence and the true population value and confidence of 95%; (2) minimum prevalence ratio estimates, which would be achieved with a test power of 80.0%, i.e., 80.0% chance to detect differences between aggressor and non-aggressor users at a level of significance of 5%, regarding the potential exposure to risk factors.

The questionnaire used was similar for women and men, being used the same questions to gather the demographic characterization and psychological, physical, and/or sexual violence suffered by women and practiced by men. To identify the perception of having suffered some violence in life, we used the same question, both for women and men. It should be noted that only in this part of the questionnaire the word violence was mentioned. The objective was to verify how the term corresponded to situations of aggression experienced by men and women^4,10^.

The variables were described using mean, standard deviation, frequencies, and proportions. To test the hypotheses, we used the Fisher’s exact and Pearson’s Chi-square tests. We adopted the significance level of 5%.

This study was approved by the Research Ethics Committee of the Medical School/Hospital das Clínicas in FMUSP on 5/12/2000 and 12/11/2002, for the research with women and men, respectively. We resorted to the informed consent form and other ethical measures recommended by the World Health Organization for sensitive issues such as violence^10^.

## RESULTS

### Sample Description

Table 1 shows that the men were older, had higher level of education and the greatest proportion were composed of black and non-catholic men.

**Table 1:** Sociodemographic characteristics of men and women in the study. São Paulo, SP, Southeastern Brazil, 2002-2003 and 2001-2002.

Variable	Gender			

	Man (n = 775)		Women (n = 661)	
Mean Ages (SD)	36.02 (11.0)		30.65 (9.1)	
p			< 0.001	
Marital State	n	%	n	%
Married	513	66.2	391	59.1
Boyfriend	121	15.6	132	20.0
No partner	141	18.2	138	20.9
p				0.019
Years of study				
0-8	454	58.6	411	62.2
9-11	243	31.3	228	34.5
> 11	78	10.1	22	3.3
p				< 0.001
Race/Color				
Black	374	48.3	237	35.8
Not black	401	51.7	424	64.1
p				< 0.001
Religion				
Catholic	429	55.3	420	63.5
Not catholic	346	44.6	241	36.5
p				0.002

### Episodes of Violence Suffered and Perpetrated

As for the violence suffered by the intimate partner in life, women showed higher frequency, regardless of the type (psychological, physical, or sexual). Regarding the perpetration of physical violence, we observed higher proportion among men (Table 2).

**Table 2:** Frequencies, proportions and perception rate of violence suffered and committed by intimate partners, according to gender. São Paulo, SP, Southeastern Brazil, 2001-2003.

Violence suffered	Sex			

	Men		Women	
	
	n	%	n	%
Psychological				
No	720	92.9	299	47.4
Yes	55	7.1	332	52.6
p				< 0.001
Physical				
No	754	97.3	395	62.6
Yes	21	2.7	236	37.4
p				< 0.001
Sexual				
No	763	98.5	503	79.7
Yes	12	1.6	128	20.3
p				< 0.001
Experienced any violence (psychological, physical, or sexual)				
No	696	89.8	247	39.1
Yes	79	10.2	384	60.9
p				< 0.001
Perpetrated any physical violence				
No	528	68.1	425	88.9
Yes	247	31.9	53	11.1
p				< 0.001
Suffered or perpetrated				
Did not Suffer or perpetrate	232	38.0	185	40.0
Just suffered	19	3.1	224	48.5
Just perpetrated	299	49.0	4	0.9
Suffered and perpetrated	60	9.8	49	10.6
p				< 0.001

By analyzing the concomitance of the violence suffered and perpetrated between men and women (Table 2), higher proportion of violence perpetration was verified among men, and of violence suffered in life, among women.

### Comparison between Intimate Partner Violence: suffered by women, according to the their report, and perpetrated by men, also according to their report and the respective perceptions of the act

When comparing reports of violence suffered by women and perpetrated by men, we found higher episodes of violence suffered, regardless of the type. This same profile remained regarding the recurrence of any type of intimate violence over the past year and in life (Table 3).

**Table 3:** Comparison between the proportions of violence by intimate partner suffered by women and perpetrated by men. São Paulo, SP, Southeastern Brazil, 2001-2002 and 2002-2003.

Types of violence	Suffered by women		Perpetrated by men	
	
	n	%	n	%
Psychological				
Yes	332	52.6	310	40.0
No	299	47.4	465	60,0
p				< 0.001
Physical				
Yes	236	37.4	247	31.9
No	395	62.6	528	68.1
p				0.003
Sexual				
Yes	128	20.3	30	3.9
No	503	79.7	743	96.1
p				< 0.001

Recurrence of violence	Suffered by women		Perpetrated by men	

A lot in the last year				
Yes	91	23.7	18	5.6
No	293	76.3	303	94.4
p				< 0.001
A lot in life				
Yes	126	32.8	28	10.1
No	258	67.2	249	89.9
p				< 0.001

Severity of violence	Suffered by women		Perpetrated by men	

In life				
No violence	277	41.9	371	47.9
Some psychological	110	16.6	310	40.0
Only moderate	69	10.4	72	9.3
At least one severe injury	205	31.0	22	2.8
p				< 0.001

Perception of violence	Women who said that suffered		Men who said that perpetrated	

Yes	190	28.8	239	30.8
No	469	71.2	536	69.2
p				0.108

Physical violence	Perpetrated by women		Suffered by men	

Yes	53	8.0	21	2.7
No	608	92.0	754	97.3
p				< 0.001

Additionally, more women reported at least one serious violent attack, while men reported greater perpetration of psychological violence (Table 3). As for the perception of the violence suffered by women and perpetrated by men, we observed that men and women had, also, low perception of violence occurrence against the acts of aggression actually experienced.

In the crossing-over reverse to the previous one, and considering only the physical violence, women reported more perpetration than men reported having suffered violence (Table 3).

Finally, we observed statistically significant differences in the comparison between the women’s perception of having suffered violence and the men’s perception of perpetration. Both presented low perception of violence, although this convergence had been modified due to the acts of greater severity and recurrence of episodes (Table 3).

### Study of the common Service for Women and Men

Regarding the comparison of intimate partner violence and their perceptions, when considering the violence suffered by women and the one perpetrated by the men interviewed in the same health service, we observed that, in physical violence, there was no statistically significant difference between the proportions of reports of suffering and violence perpetration. Psychological and sexual violence, however, kept the same profile of the total sample of men and women, i.e., greater proportion of reports of suffering by women (Table 4).

**Table 4:** Violence cross-linked to the reports of women and men among users of the same service. Center, São Paulo, SP, Southeastern Brazil, 2001-2003.

Violence	Suffered by women (n = 325*)		Perpetrated by men (n = 388)	
		
Types of acts	n	%	n	%
Psychological				
Yes	176	55.5	164	42.3
No	141	44.5	224	57.7
p				< 0.001
Physical				
Yes	129	40.7	131	33.8
No	188	59.3	257	66.2
p				0.06
Sexual				
Yes	63	19.9	18	4.6
No	254	80.1	370	95.4
p				< 0.001

Recurrence of acts	Suffered by women (n = 207)		Perpetrated by men (n = 213)	

Once	73	35.2	33	15.3
A few times	71	34.3	162	76.3
Many times	63	30.5	18	8.4
p				< 0.001

Perception of violence	Women who said that suffered (n = 324)**		Men who said that perpetrated (n = 388)	

Yes	97	29.9	112	28.9
No	227	70.1	276	71.1
p				0.754

Physical violence	Perpetrated by women (n = 325)		Suffered by men (n = 392)	

Yes	31	9.5	9	2.3
No	294	90.5	383	97.7
p				< 0.001

* 8 missings.

** 1 missing.

Finally, we observed statistically significant differences in the comparison between the women’s perception of having suffered violence and the men’s perception of perpetration. Both present low perception of violence, which changes when considering the recurrence of episodes (Table 4).

Finally, when it came to the crossing-over on physical violence suffered by men and perpetrated by women, we observed that the women reported higher perpetration than men reported having suffered physical violence (Table 4).

## DISCUSSION

Victimization is greater among women, whether psychological, physical, or sexual, regarding violence perpetrated by intimate partners. This finding is very discussed in the literature^5,11,16^ that deals with studies of violence suffered by women and by men as independent investigations, as earlier pointed out, but is also a finding of those studies that address women and men in the same investigation. Selic et al.^17^ observed higher prevalence of physical and psychological violence among female users of healthcare unit in Slovenia. In study conducted with 762 couples in divorce mediation process, women showed the highest frequency of all types of violence examined (psychological, sexual assault, physical threat, and coercion), except for physical abuse, for which the difference between genders was not identified^18^. The highest prevalence of intimate partner violence among women, regardless of the type, is consistent with the population survey with a representative sample of the Brazilian population^19^.

All these studies are related to violence by intimate partners, situation that the literature points out as much less frequent for men than for women. As a result, when you consider the violence men suffer by any aggressor, the frequency of episodes exceeds the women’s, especially in the case of physical violence^4^. Thus, in general terms, men are the biggest victim of aggression, but in terms of domestic violence, women occupy this position. However, when examining sexual violence in particular, whose prevalence is less frequent than physical or psychological violence, for both genders, women are the biggest victims, whether in the household context or not. This specificity is a characteristic of gender inequality, which is reinforced by the meaning quite distinct between physical and sexual aggression. In Brazilian study with users of health services^20^, the term violence was recognized by women who suffered this type of aggression (70.0%) as being more associated with situations of sex forced by the intimate partner, than associated with situations of physical aggressions by the partner (47.0%). That means that the term violence, which refers to the violation of rights and implies serious situation of aggression, is more related to sexual violence, in Brazilian culture.

Another very important gender issue concerns the overlap of the violence suffered with the aggressions perpetrated, in the case of men^16^. Brazilian study^4^ indicates approximately 90.0% of overlap, hindering the analysis about associated factors and the impacts of suffering violence on health. We noted that in this study, as it is only about the situation of intimate partner violence, men were characterized by their condition of perpetrators, especially. Thus, women were little characterized as perpetrators of violence, in this situation of intimate relationship.

On the other hand, this finding differs from international studies, in which no difference was observed between prevalence of violence suffered, regardless of the type, between men and women^11,19^, indicating a possible gender symmetry in perpetration and victimization. Another study conducted with young student couples, held in Mexico, also found that domestic victimization is similar between men and women^16^. However, especially in the case of young people, the possible gender symmetry makes us think that the profile of violence suffered and perpetrated changes with age or time of the relationship. This was one of the possible explanations raised by the Brazilian study with university students who were dating, in which we could not observe differences of violence suffered and perpetrated between men and women^21^.

It is important to remember that the various studies mentioned used different methodologies, which may induce different understandings about the violent acts suffered or perpetrated. Additionally, the research conducted with couples, different from this study, allow better analysis of gender symmetry, but may result in overestimation of the revelation of violence in one of the partners, due to retaliation.

One last point to consider is the exam that was conducted about the perception of violence suffered by women and perpetrated by men. As our findings show, that apparent gender symmetry is relativized, when considering the low rates of violence broken down by the types of acts and recurrence of episodes. Corroborating with international study^14^, our data, when showing that women report episodes of multiple recurrences of any violence and sexual assault suffered more than men acknowledge to have perpetrated, suggest a clear distinction of gender. We are led to interpret these findings as a low recognition of men regarding the aggression perpetrated by them, in accordance with the trivialization of violence in intimate relationships. In gender studies, this trivialization is explained by the fact that for men, violence against their partner corresponds to the correct way to act, which is historically constructed and valued, to solve conflicts and to acculturate the woman, i.e., to put her in a subordinate role in this intimate relationship^22^.

Particularly regarding sexual violence, perceived by women but not by men, it is noteworthy the social construction of women’s obligation towards the sexual relationship in the marriage contract that accompanies the intimate relationship. In addition to sex against the women’s will or with absence of desire, sometimes coercive and derogatory sexual practices occur, which are justified by the obligation with the male pleasure. Other times these acts are interpreted by the men as desire, despite the women’s explicit refusal.

When considering the internal validity of the study, its achievement in independent health units, with interviews conducted at different times, aimed to minimize answers that could cause retaliation. In addition, the profile of perpetration and victimization of intimate partner violence was confirmed with analysis of the subsample of men and female users of the same health service, which enhance the validity of the findings. One limitation of the study, however, is the difference of two years between the period of data collection of men and women. Nevertheless, this time lag did not present major changes in the dissemination of research about violence by intimate partners or even a great treatment by the media, as it can be seen after 2005. Neither there was public policy changes relating to domestic violence, which could interfere with health services, implying the users. Important to point out that, although data collection in both health units occurred before the Maria da Penha Law^a^, our findings have enabled the discussion with national and international studies with different methodologies, which reinforces the importance of this study under the analysis of gender symmetry. Thus, we conclude that the study in its entirety shows significant gender differences, whether about the prevalence of violence, whether about the perception of these situations.
